# Influence of an increased number of physical education lessons on the motor performance of adolescents–A non-interventional cohort study

**DOI:** 10.1371/journal.pone.0258305

**Published:** 2021-10-14

**Authors:** Astrid Reif, Christoph Triska, Michael Nader, Jürgen Scharhag, Harald Tschan, Barbara Wessner

**Affiliations:** Department Sports Medicine, Exercise Physiology and Prevention, Institute of Sport Science, Centre for Sport Science and University Sports, University of Vienna, Vienna, Austria; University of Pennsylvania, UNITED STATES

## Abstract

Increasing the amount of regular physical education lessons in school is currently discussed in many countries in order to increase physical activity in youth. The purpose of this study was to compare the motor performance of pupils from an observation group participating in a school trial of two additional physical education lessons (5 lessons of each 50 min/week) without a specific intervention program to a control group with a regular amount of three physical education lessons (3 lessons of each 50 min/week) as indicated by the standard Austrian school curriculum. In this cohort study motor performance of 140 adolescents (12.7±0.5 years) was assessed by means of the German Motor Performance Test 6–18 over a period of 1.5 years with measurement time points before (T1), after eight months (T2) and at the end of the observation period (T3). Two- and three-way mixed analysis of variance were used to detect time, group and interaction effects. Although the observation group demonstrated a higher total motor performance score at all time points (P = 0.005), the improvement over time in total motor performance (P < 0.001) was more pronounced in the control group. Girls and boys developed differently over time (time*gender interaction: P = 0.001), whereby group allocation did not affect this interaction (time*gender*group: P = 0.167). Anyway, girls of control group tend to benefit most of additional physical education lessons. Sports club members scored significantly higher in motor performance across the observation period (P = 0.018) irrespective of group allocation. These findings indicate that there could be a ceiling effect in what the pupils could achieve in terms of motor performance as the pupils of the observation group might have reached this point earlier than their counterparts in the control group. Nevertheless, sports club membership seems to reveal some benefits. Whether improving quality and specificity of the single physical lessons might be superior to merely adding additional ones needs to be confirmed in future studies.

## Introduction

Particularly in young people there is a global trend that the majority does not meet current physical activity guidelines [1]. Therefore, daily or increased amount of physical education (PE) lessons, is a common practice to extend movement time in daily school life [2–4] as they are able to reach pupils through all social classes [5]. School-based intervention-programs have demonstrated positive influence on health parameters [5, 6] and also on motor performance [7, 8]. The temporal development of motor performance in youth is a dynamic process influenced by personal characteristics but also environmental context such as physical activity or socio-economic status [9]. Firstly, body composition is suggested to have the biggest influence on the temporal development of motor performance as a high amount of fat percentage goes along with a weaker development of motor performance during maturation [10]. Secondly, a certain relationship is also known between motor performance and age [11]. Especially in adolescence there is a prevalence to clumsiness and growth spurts because frequently, body size accelerates more rapidly than the capacity to adapt motor performance and they are more likely to dropout from being frequently physical active [12]. Thirdly, gender differences in motor performance are merely detected at a younger age [13], but at the beginning of adolescence boys accelerate their development and pass girls in many fundamental motor abilities [9]. Additionally, to physiological causes, behavioural differences might contribute to gender differences as lesser engagement in physical activity is observed in adolescent girls [11] which directly contributes to motor performance [10, 11, 14]. An important point to consider is that pupils might compensate a higher amount of PE lessons by lesser physical activity during leisure time, anyway previous results are controversial [4, 15, 16]. It stays unclear whether certain extracurricular activities would affect motor performance. At the age of thirteen years the so-called “lifelong-utilization-stage” according to Ozmun and Gallahue [17] is reached. Motor performance has reached a peak at this stage and therefore determines lifelong utilization in daily living, recreational, and competitive utilisation [17].

In this research the global term motor performance is used to reflect fundamental movement skills including the skill of performing them in a testing situation. Together, this indicates that there is a variety of influences which must be considered when analysing the development of motor performance.

Previous studies in school-settings evaluated motor performance of youth having additional, specially designed training programs. For example, a significantly higher development of motor performance has been demonstrated in an intervention study with PE lessons five times per week with specific training program compared to a control group (CG) with only two PE lessons per week [7, 8]. Furthermore, when comparing two different school-based intervention programs adolescents in both intervention groups benefited from the structured training program compared to the controls. Further, subpopulations are differently affected by school-based physical activity interventions. Previous results show that cardiorespiratory fitness of girls and older students benefitted to a higher extent compared to boys and younger students [18]. In summary, PE intervention programs show some benefits, but they often lack on sustainability as implementing them into the everyday school setting seems to be cumbersome. Previous studies provided evidence that school-intervention programs can have a benefit on pupils’ motor performance. It stays still unclear whether additional PE lessons without specific training program may lead to the same effect. The real-world scenario of the present study is a benefit to evaluate the motor performance of additional PE lessons without a specific intervention program.

Therefore, the aim of the study was to assess whether an increased number of PE lessons would have an influence on motor performance of adolescents at the age of 12 to 14 years. Secondary analysis addressed the impact of gender, extracurricular participation in sports clubs and movement behaviour outside school on motor performance.

## Materials and methods

### Study design

This observation study without any intervention assesses the development of motor performance of adolescents with two additional PE lessons weekly. Participants of CG had a regular amount of three PE lessons per week, whilst participants of observation group (OG) had five. Participants’ motor performance has been observed three times over the course of 1.5 years (three school semesters). The assessment took place at the beginning (T1), eight months after beginning of the observation period (T2), and at the end of the study (T3). The study was approved by the Ethics Committee of the University of Vienna (#00236). All procedures were performed in accordance with the ethical standards of the Helsinki Declaration as revised in 2013. The study is reported as predetermined in the STROBE reporting guidelines for cohort studies [19] and registered retrospectively in the German Register of Clinical Trials at the Clinical Trials Registry Platform of the WHO (DRKS00022241).

### Setting and selection of schools

Schools were selected in a way that OG and CG had a similar setting of infrastructure and urban surroundings, to be distinguished only in the difference with respect to the number of PE lessons. In October 2016, the observation school was chosen by the criterion to have five PE lessons (250 min) per week. Control schools were characterized by offering three PE lessons (150 min) per week and should ensure comparable infrastructure and urban surroundings to minimize socio-economic influences, which are well known to influence physical activity time [5, 9]. Therefore, control schools were selected arbitrarily within the same urban region (Linz/Austria, population about 207.000 people). None of the schools had a specialisation in sports or an entrance examination in sports to minimize a potential selection on special interests or talent in sports. A special emphasis was put to observe an already existing school trial to raise PE lessons without interrupting teaching content or structure. Teachers of OG and CG were planning and conducting PE lessons within the same curricular framework which was the Austrian standard curriculum. The Austrian standard curriculum has the aim to develop condition and coordination abilities while promoting enjoyment in sports. Commitment to performance but also to lifelong physical activeness should be part of the teaching contents. The curriculum is divided in six parts of movement fields: Basic movement skills, competence- and performance-oriented movements, playful activities, creative movements and performing arts, health-oriented and compensatory movements, and experience-oriented movements [20]. This governmental standard curriculum provides the framework for the content as well as the amount of PE lessons of CG. All schools gave written consent for participation by the school principal.

### Participants

**Fig 1:**
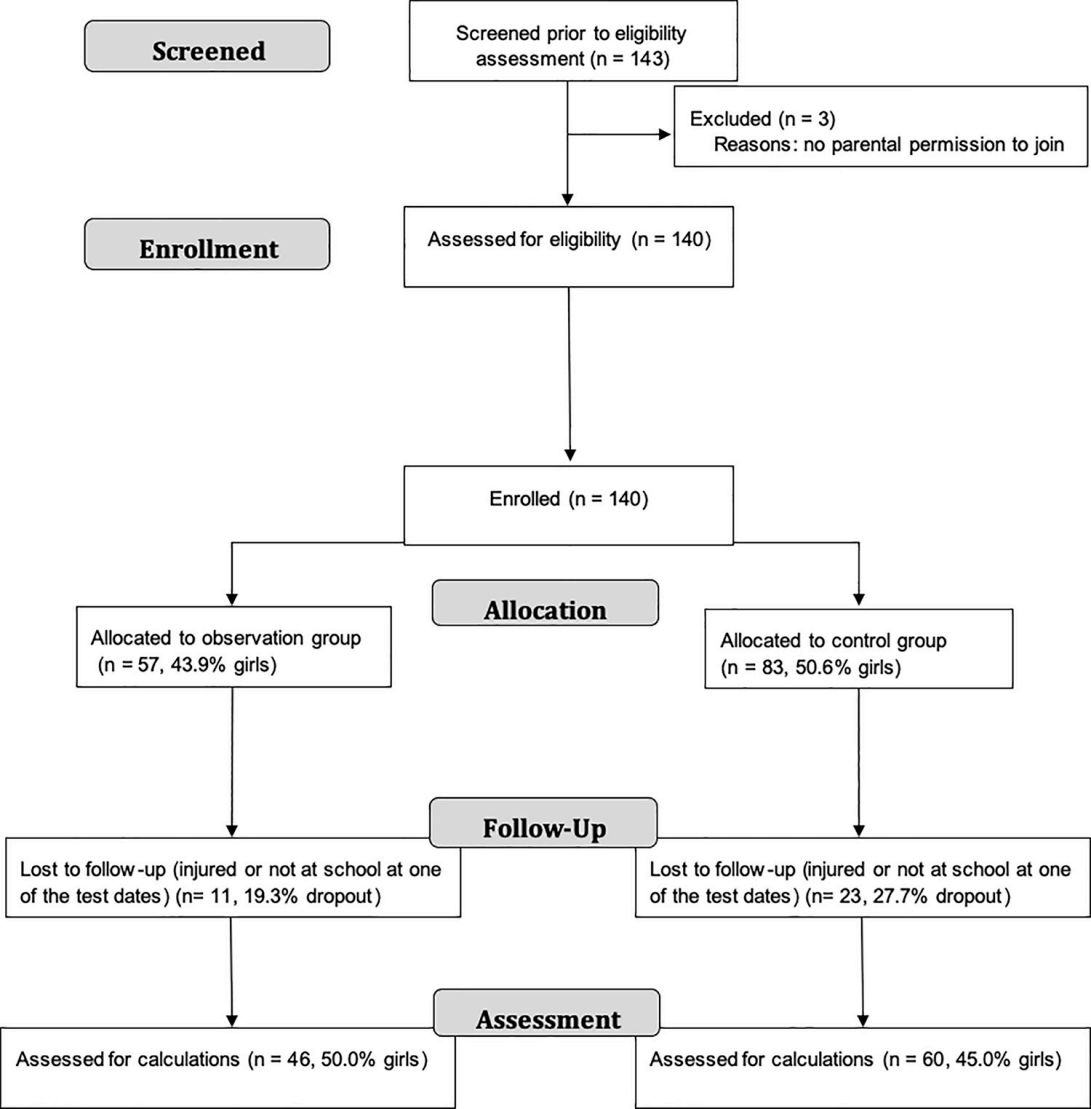
Participants. Consort flowchart demonstrating gender distribution and dropout numbers.

The exposure of two additional PE lessons of the OG was limited to the seventh and eight school level. Therefore, inclusion criteria for voluntary participation in the study was to attend the seventh grade of the observation or control schools to ensure a follow up to the eighth grade. Further, they had to participate actively at the PE lessons of each data collection time point. Participants being absent, sick, or injured at one of the three data collection dates were excluded from the analysis. Also, participants missing more than 80% of PE lessons during the whole observation time would have been excluded, which was not the case. To volunteer for this study, participants as well as their legal guardian had to give declaration of consent in written form. Based on these criteria, 140 pupils were initially recruited and 106 of those could be included in the final analysis. Detailed information of the participants flow is presented in Fig 1 and demographic and anthropometric data at baseline are presented in Table 1.

**Table 1:** Descriptive data for demographic and anthropometric characteristics.

*Characteristics of baseline sample*	*Total (n = 140)*	*OG (n = 57)*	*CG (n = 83)*	*p value*
Age (years)	12.69 ± 0.54	12.67 ± 0.55	12.71 ± 0.53	0.633
Body stature (m)	1.62 ± 0.08	1.62 ± 0.08	1.63 ± 0.08	0.470
Body mass (kg)	50.34 ± 9.80	49.51 ± 10.04	50.91 ± 9.65	0.407
BMI (kg/m^2^)	19.05 ± 2.82	18.84 ± 2.55	19.19 ± 3.00	0.479
*Temporal change (ΔT3-T1)*	*Total (n = 106)*	*OG (n = 46)*	*CG (n = 60)*	*p value*
Body stature (m)	0.06 ± 0.04	0.06 ± 0.04	0.07 ± 0.04	0.742
Body mass (kg)	6.36 ± 4.67	5.99 ± 4.30	6.65 ± 4.95	0.469
BMI (kg/m^2^)	0.77 ± 1.19	0.75 ± 1.04	0.79 ± 1.30	0.852

Data are expressed as means ± standard deviation or absolute numbers to determine differences between OG and CG independent t test and ^1^chi^2^ have been used; P < 0.050 was considered statistically significant.

BMI = body mass index, m = meters, kg = kilograms.

### Assessment of motor performance

Motor performance was assessed using the German Motor Performance Test 6–18 (GMT) [21] which represents a commonly applied test validated for children and adolescents aged between 6 to 18 years. The assessment of motor performance consists of eight exercises including a 20-m sprint, sit-ups, push-ups, standing long jump, balancing backwards (on wooden beams of different broadness), jumping from side to side, 6-min run, and forward bend from a standing position.

Prior to the tests demographic and anthropometric data such as body stature, body mass, date of birth and gender were assessed. A total score variable of motor performance was calculated by the average score of the exercises (despite balancing backwards, which is interpreted separately as it is seen as a limiting factor but not as an alone standing motor ability) [21].

Motor performance was assessed in the gym of the respective school during PE lessons in order to provide a familiar environment for the pupils. A standardized 10-min warm-up program consisting of mobility exercises and slow jogging was performed prior to all tests. To ensure similar testing conditions, the tests were applied at the same time of the day and the same examiners assisted by the PE teachers carried out the tests throughout the study. Pupils were familiarized with the procedures and starting positions for each test were standardized according to the instructions of the test. Exercises were performed in a random order, but with speed tested at the beginning and endurance at the end of the test battery. The variables were calculated using z-scores for each single test exercise and Z-scores for total scores. These scores are sex- and age specific standardised values calculated based on a German reference sample. The calculation of the z-scores is described in the manual [21, 22]. The normal range for z- and Z-scores is set between 95.00 and 104.99. A score of 100.00 represents average motor performance compared to the German sample with similar background [22]. To ensure accurate measurements the timing system was a sprint single-beam timing gate (Witty-Gate, Microgate, Bolzano, Italy) as it provides a more accurate measurement compared to stopwatches [23]. For the subsequent calculations, 0.2 s of reaction time were added to enable a comparison with the reference data given by the authors of the battery. Reliability, validity, objectivity and the potential to observe the development of youth have been demonstrated for the GMT 6–18 [21].

### Assessment of sports club membership and physical activity outside school

In order to assess a potential influence of sports club membership on motor performance, pupils were asked for sports club membership by a questionnaire at each data collection time point (spring, autumn, summer). Pupils were assigned to the sports club membership group if they reported being a member in at least one sports club in two different time points, while all other pupils (those who did not participate in sports clubs and those who reported being a member in only one time point) were assigned to the non-sports club membership group. Additionally, we assessed physical activity outside school by a questionnaire to ensure comparable groups. Children were asked, if they were able to fulfil the daily movement recommendations by the World Health Organization of 60 min moderate to vigorous physical activity [24]. Moderate to vigorous physical activity has been defined as sweating and/or accelerated breathing. These data were assessed excluding physical activity time in PE lessons and sports clubs and were collected for school days and school-off days. Following two questions assessed this variable: “In addition to physical education and training hours in sports clubs: How often are you physically active for at least 60 minutes so you have to breathe faster or sweat?” and “How often are you physically active for at least 60 minutes so you have to breathe faster or sweat on days off from school?”. For both questions children could choose between the answers (1) everyday, (2) 3–4 times per week, (3) 1–2 times per week, or (4) never.

### Bias

To avoid a possible bias through circumstances of school-settings, control schools have been chosen carefully (as described in the methods) to enable a comparable sample regarding socio-economic surroundings and infrastructure. The CG was recruited from two different schools because selecting homogenous groups of the target population but from different institutions may strengthen the studies’ external validity [25]. Therefore, similar school-settings between groups could be ensured despite different exposures (amount of PE lessons).

Due to the observational character of the study differences in teaching content and quality are unavoidable. However, teaching had been conducted and planed by teachers who were educated in a similar way at one of the four Austrian universities that offer a study programme in PE. The teaching content was limited to the curricular framework defined by the government [20]. Furthermore, PE contents were listed by the teachers and have been compared by the principal investigator to confirm the compliance with the standard curriculum. Still, there was some flexibility in content. Therefore, PE teachers were asked to fill in a questionnaire of a ranking on the focus of their teaching with respect to the main assessment variables of the DMT (speed, strength, coordination, aerobic endurance, and flexibility).

Growth spurts are common during adolescence and might affect motor performance [12]. To avoid this bias, development of body stature, body weight and BMI through observation period have been assessed.

Further, a possible bias might be a period of 9 weeks of summer holidays. However, the data collection in summer happened before the holidays and the one in autumn already two months after school has started, so children were used to their PE schedule in all data-collection points. Furthermore, this bias would have affected both groups in the same conditions.

### Sample size

OG had higher amount of PE lessons based on a school trial. No other school in the close, comparable area was fulfilling the selection criterion of five PE lessons (250 min) per week and using the Austrian standard curriculum. To enable a high number of participants in OG, all participants of the 7^th^ school year (three classes, n = 57) were invited to participate to the study. The CG was formed from two schools (two classes each, n = 83) to achieve a similar-sized control. Therefore, sample size was a priori defined by the number of available students in the OG rather than by sample size estimation. However, a post hoc analysis on the main outcome (total performance Z-score) using G*Power 3 [26] revealed a statistical power (1 –β) given an α level of 0.05, a sample size of n = 106, a calculated effect size f of 0.35, and the observed partial eta^2^ of 0.11 for the group x time interaction effect which denotes the primary research question of 1.00.

### Statistical analysis

According to the central limit theorem the distribution of sample means approximates a normal distribution, as the sample size becomes larger [27]. Therefore, normal distribution of metric variables was assumed as sample size was n ≥ 30 for all comparisons. Independent t-tests (using Levene´s test and correction to analyse and correct equality of variances) were used to compare groups at baseline, whereas a two-way mixed analysis of variance (ANOVA) served to detect differences in the development of motor performance at time points T1-T3, between groups and corresponding interactions. Subgroups were examined through a three-way mixed ANOVA. Violations of sphericity were considered by Greenhouse-Geisser corrections. Post-hoc calculation was conducted for time effects using Bonferroni corrections. In case of significant time*group interactions simple main effects for time separated by groups were calculated using Bonferroni corrections. Partial eta-squared ($\eta_{p}^{2}$) was used to provide an estimate of effect size of the ANOVA (trivial $\eta_{p}^{2}$ < 0.01; small 0.01 ≤ $\eta_{p}^{2}$ < 0.10; moderate 0.10 ≤ $\eta_{p}^{2}$ < 0.25; large $\eta_{p}^{2}$ ≥ 0.25) and Cohens d was applied for effect sizes in t-tests (small 0.02 ≤ d < 0.5; moderate 0.5 ≤ d < 0.7; large d ≥ 0.8) [28]. For comparison of categorical data between groups a chi^2^ (χ^2^) test and Man-Whitney U-Test was applied. For all tests the alpha level of statistical significance was set a priori at P < 0.050. When data was missing (absence or sickness of participants) the participants’ results were excluded from final analysis resulting in a per protocol analysis of data. The analysis were conducted using SPSS statistical software package 25 (IBM SPSS Statistics, SPSS Inc., Chicago, USA). All data is presented as mean ± standard deviation or as absolute, relative frequencies and median in case of nominal or ordinal data.

## Results

### Sample

At the baseline of the study, no significant differences between OG and CG were found for age, body stature, body mass and BMI in the total study population (n = 140, see also Table 1). Detailed information for dropout numbers and gender distribution is presented in Fig 1. There were no differences in dropouts between groups nor in gender (χ^2^_1,106_ = 1.30, P = 0.254; χ^2^_1,106_ = 0.83, P = 0.774). Gender distribution did not differ significantly in the final sample (47.2% female; χ^2^_1,106_ = 0.26, P = 0.609) neither in OG (50.0% female; χ^2^_1,46_ = 0.00, P = 1.00) nor in the CG (45.0% female; χ^2^_1,60_ = 0.60, P = 0.439). In the final sample, no significant differences between OGs and CGs anthropometric development over time have been found for body stature, body mass, and BMI.

### Motor performance at baseline

Significant differences in total score of motor performance were found for baseline testing between groups represented by a large effect size (t_135.22_ = 4.83, P < 0.001, d = 0.83). Thereby, six out of eight exercises demonstrated significant differences with higher scores in OG (balancing backwards (t_137.87_ = 6.55, P < 0.001, d = 1.12), jumping from side to side (t_134.47_ = 3.55, P = 0.001, d = 0.61), push-ups (t_138_ = 2.35, P = 0.020, d = 0.40), sit-ups (t_138_ = 3.26, P = 0.001, d = 0.56), 6-min run (t_137.55_ = 2.81, P = 0.006, d = 0.48)). However, no significant differences were observed in sprint (t_138_ = -0.20, P = 0.842, d = 0.03) and forward bend (t_138_ = -1.62, P = 0.109, d = 0.28).

### Elevated amount of lessons and motor performance

**Fig 2:**
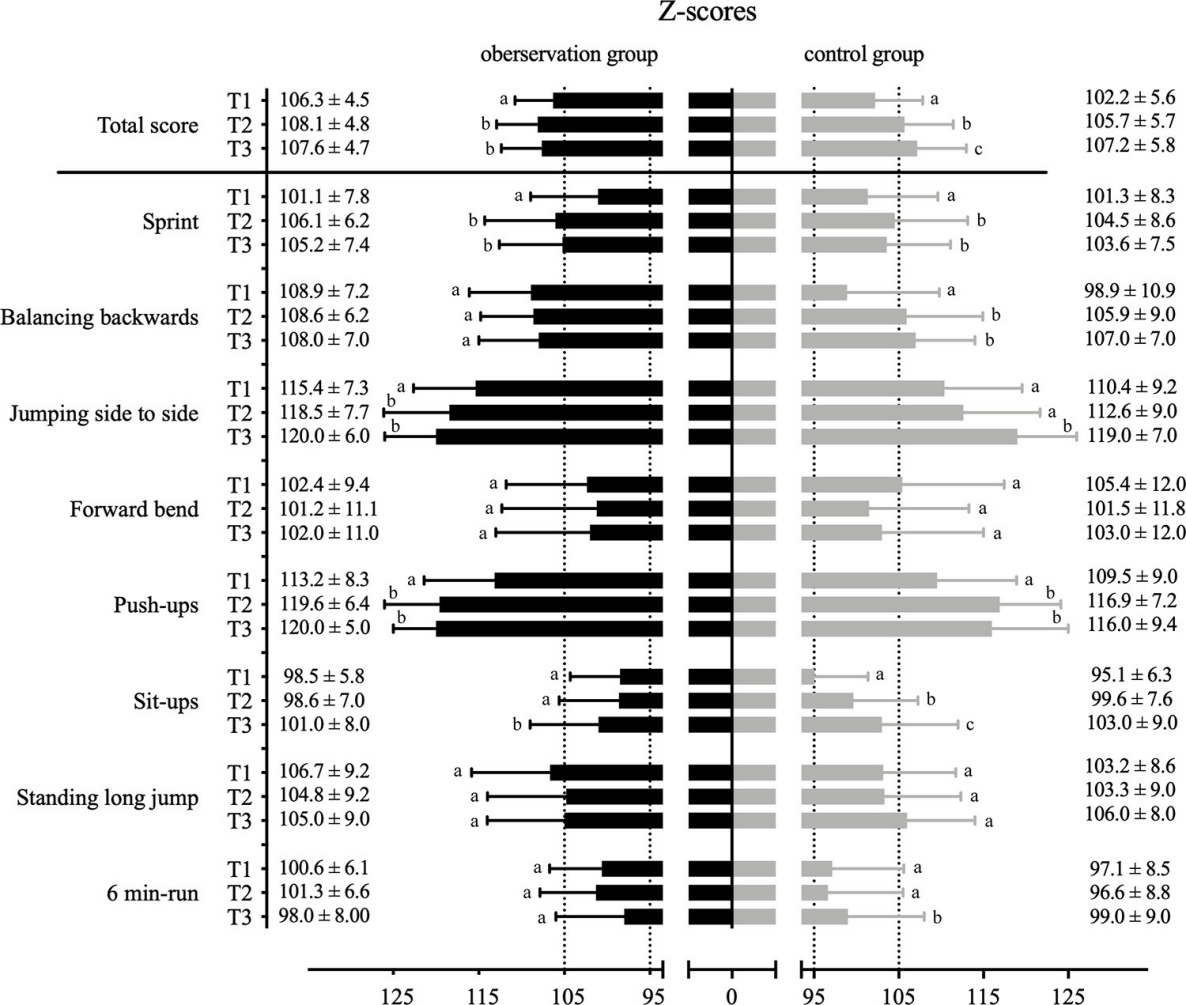
Z-scores of motor performance comparing observation and control groups at three time points. Data represents motor performance comparing means ± standard variations of OG and CG at three time points (T1-T3), calculated in Z-scores (total score) and in z-scores (individual exercises), normal range is marked by the dashed lines (N = 102–106). Letters refer to Bonferroni-corrected post hoc analysis of potential time effects, whereby different letters refer to significant differences between time points (P < 0.050).

Individual test scores for T1-T3 are presented in Fig 2 and corresponding P-values and effect sizes are shown in Table 2.

**Table 2:** Z-scores of motor performance considering all three time points.

*Variables*	*Effects*	*F*	*df*	*p*	*partial η^2^*
Total score	Effect of time	54.74	2.00	<0.001	0.35^L^
(n = 106)	Effect of groups	8.18	1.00	0.005	0.07^S^
	Effect of interaction (time*group)	13.09	2.00	<0.001	0.11^M^
Sprint	Effect of time	22.79	1.83	<0.001	0.18^M^
(n = 105)	Effect of groups	0.33	1.00	0.568	0.00^T^
	Effect of interaction (time*group)	1.26	1.83	0.285	0.01^S^
Balancing backwards	Effect of time	13.76	1.67	<0.001	0.12^M^
	Effect of groups	15.77	1.00	<0.001	0.13^M^
(n = 106)					
	Effect of interaction (time*group)	12.87	1.67	<0.001	0.11^M^
Jumping from side to side	Effect of time	51.31	2.00	<0.001	0.34^L^
	Effect of groups	16.91	1.00	<0.001	0.15^M^
(n = 102)	Effect of interaction (time*group)	11.66	2.00	<0.001	0.10^M^
Push-Ups	Effect of time	46.07	1.82	<0.001	0.31^L^
(n = 105)	Effect of groups	9.00	1.00	0.003	0.08^S^
	Effect of interaction (time*group)	0.24	1.82	0.769	0.00^T^
Sit-Ups	Effect of time	45.43	1.96	<0.001	0.30^L^
(n = 106)	Effect of groups	0.68	1.00	0.412	0.00^T^
	Effect of interaction (time*group)	10.05	1.96	<0.001	0.09^S^
Standing long jump	Effect of time	3,47	1.89	0.033	0.03^S^
	Effect of groups	0.80	1.00	0.374	0.01^S^
(n = 105)	Effect of interaction (time*group)	4.44	1.89	0.013	0.04^S^
6-min run	Effect of time	0.94	1.82	0.385	0.00^T^
(n = 101)	Effect of groups	4.70	1.00	0.033	0.05^S^
	Effect of interaction (time*group)	1.20	1.82	<0.001	0.09^S^
Forward bend	Effect of time	2.52	1.44	0.101	0.02^S^
(n = 106)	Effect of groups	0.27	1.00	0.607	0.00^T^
	Effect of interaction (time*group)	1.16	1.44	0.303	0.01^S^

Data represents two-way repeated measure ANOVA of total score; ^T^ = trivial effect size; ^S^ = small effect size; ^M^ = moderate effect size; ^L^ = large effect size, (n differs between exercises as some children did not attend all exercises, if they felt pain doing a specific movement due to an old injury).

For the total score significant time, groups and time*groups interaction effects have been found. Bonferroni-corrected post-hoc tests revealed significant differences between time points in the total sample (T1-T2: P < 0.001; T2-T3: P = 0.011 and T1-T3: P < 0.001) and separately for CG (all at P < 0.001) and in OG in T1-T2 and T1-T3 (both at P = 0.002).

For 20-m sprint a significant effect of time has been found with significant post-hoc differences between T1-T2 and T1-T3 (both at P < 0.001). Balancing backwards demonstrated significant time, groups and time*groups interaction effects with significant differences revealed by post-hoc calculation in total sample between T1-T2 (P = 0.001) and T1-T3 (P < 0.001) and in CG between T1-T2 and T1-T3 (both at P < 0.001). For jumping from side to side significant time, groups and in time*groups interaction effects have been found. Bonferroni-corrected post-hoc tests revealed significant differences between all time points in the total sample (T1-T2: P = 0.001; T2-T3 and T1-T3 both at P < 0.001). Significant differences have been found in OG between T1-T2 and T1-T3 (both at P < 0.001) and in CG between T2-T3 and T1-T3 (both at P < 0.001). For push ups significant time and groups effects have been found with significant differences in post-hoc tests between T1-T2 and T1-T3 (both at P < 0.001). For sit ups significant time and time*groups interaction effects have been found and Bonferroni-corrected post-hoc tests demonstrated significant differences between all time points in the total sample (all at P < 0.001). Significant differences have been found in OG between T2-T3 (P = 0.003) and T1-T3 (P < 0.001) and in CG between all time points (all at P < 0.001). The 6-min run differed between groups with a significant time*groups interaction. Bonferroni-corrected post-hoc tests demonstrated significant differences in CG between T2-T3 (P = 0.042) and T1-T3 (P = 0.005), while no significant changes were observed for OG. No significant effects between time points have been detected for forward bend and standing long jump.

### Influence of gender

Descriptive data for boys and girls is presented in Table 3. Total score was not significantly different between genders (F_1,102_ = 1.01; P = 0.317, $\eta_{p}^{2}$ = 0.01). Time*gender effect was significantly different represented by a small effect size (F_2,204_ = 9.01; P < 0.001, $\eta_{p}^{2}$ = 0.08) whereby performance of the girls increased to a higher extent than those of the boys (see Table 3). Bonferroni-corrected post-hoc analysis revealed significant differences between all time points (T1-T2: P < 0.001, T2-T3: P = 0.027 and T1-T3: P < 0.001). No significant interactions were detected for gender*groups (F_1,102_ = 0.453; P = 0.502, $\eta_{p}^{2}$ < 0.01) and time*gender*groups (F_2,204_ = 1.822; P = 0.167, $\eta_{p}^{2}$ = 0.02), respectively.

**Table 3:** Motor performance score separated by gender and groups.

	*total*		*OG*		*CG*	
	*girls (n = 50)*	*boys (n = 56)*	*girls (n = 23)*	*boys (n = 23)*	*girls (n = 27)*	*boys (n = 33)*
time point 1	103.75 ± 5.42	104.27 ± 5.51	106.34 ± 4.32	106.95 ± 4.24	101.55 ± 5.36	102.40 ± 5.60
time point 2	107.89 ± 4.57	106.01 ± 6.46	108.73 ± 3.86	108.04 ± 3.86	107.17 ± 5.05	104.60 ± 6.55
time point 3	109.06 ± 4.31	109.06 ± 4.53	108.87 ± 3.81	108.00 ± 4.73	109.22 ± 5.13	106.13 ± 6.53
Δ time point 3–1	5.31 ± 4.31	5.31 ± 4.31	2.53 ± 3.26	1.05 ± 3.24	7.67 ± 3.65	3.73 ± 3.48

Data represents total score of motor performance comparing means ± standard variation (N = 102–106).

### Sports club membership and physical activity outside school

Descriptive data for sports club membership is presented in Table 4. There was no significant difference in sports club membership between OG and CG (χ^2^_1, 106_ = 0.21, P = 0.885). Irrespective of time points, total score for participants with sports club membership was significantly higher (F_2,105_ = 5.87, P = 0.018, $\eta_{p}^{2}$ = 0.05). No significant time*sports club membership (F_1,105_ = 2.50, P = 0.090, $\eta_{p}^{2}$ = 0.02) and time*sports club membership*groups (F_2,204_ = 0.08; P = 0.927; $\eta_{p}^{2}$ < 0.01) interactions were found.

**Table 4:** Motor performance score separated by sports club membership and groups.

	*total*		*OG*				*CG*	
	*sport club*	*no sport club*		*sport club*	*no sport club*	*sport club*		*no sport club*
	*(n = 66)*	*(n = 40)*		*(n = 29)*	*(n = 17)*	*(n = 37)*		*(n = 23)*
time point 1	105.23 ± 4.54	101.92 ± 6.20		107.71 ± 3.33	104.82 ± 5.06	103.41 ± 4.49		99.78 ± 6.19
time point 2	107.67 ± 4.82	105.63 ± 6.79		109.06 ±4.22	107.24 ± 5.91	106.57 ± 5.02		104.43 ±7.26
time point 3	108.57 ± 4.83	106.84 ± 6.07		109.03 ± 3.53	107.41 ± 5.26	108.21 ±5.67		106.41 ± 6.69
Δ time point 3–1	3.28 ± 4.19	4.91 ± 3.94		1.33 ± 3.23	2.59 ± 2.58	4.81 ± 4.01		6.63 ± 3.93

Data represents total score of motor performance comparing means ± standard variations.

On school days no significant difference was found between OG and CG in terms of fulfillment of the 60 min physical activity recommendation at T1 (median in OG: 3–4 x/week; median in CG: 1–2 x/week; U(46,59) = 1110.5, P = 0.087), at T2 (median in OG and CG: 3–4 x/week; U(42,57) = 1099.0, P = 0.455), and at T3 (median in OG: 1–2 x/week; median in CG: 3–4 x/week; U(45,58) = 1109.5, P = 0.169). Also on school-off days no significant differences were found between OG and CG concerning the fulfilment of 60 min physical activity recommendations at T1 (median in OG and in CG: 1–2 x/week; U(46,59) = 1315.0, P = 0.773), at T2 (median in OG: 3–4 x/week; median in CG between: 1–2 x/week and 3–4 x/week; U(41,58) = 976.0, P = 0.106) and at T3 (median in OG and CG: 1–2 x/week; U(45,59) = 1238.5, P = 0.538).

## Discussion

The present study has revealed that increasing the number of PE lessons did not result in a higher development of motor performance in pupils aged between 12 and 14 years. To our knowledge this is the first study presenting data from observing a school trial implementing a higher amount of PE lessons. Interestingly, the OG demonstrated notably higher total scores across all time points compared to CG, while temporal changes of total score in the CG outreached those of the OG. Irrespective of the number of PE lessons, adolescents with a sports club membership demonstrated significant higher motor performance scores.

To sum up, both groups notably increased their motor performance over time. At baseline, children reached higher values compared to age- and gender-related normative values, but both groups were able to further improve their scores of numerous single exercises. Having a closer look on the single exercises, it became evident that participants of the CG improved in four exercises while children of the OG only did in two which comprised jumping from side to side and sit-ups. Interestingly, in jumping from side to side OG and CG started from values higher than normative values, while in sit-ups both groups were able to catch up, with baseline values lower than normative references. In balancing backwards and the 6-min run only CG significantly improved. In both exercises, CG had slightly lower baseline values compared to normative values. Interestingly, no significant decrease of motor performance has been found, neither in total sample nor in each group separated nor in parts of the observation period (for example T1-T2 or T2-T3). Our findings were somewhat unexpected as it has been reported previously that motor performance can be increased with increasing the number of PE lessons [7]. It could be argued that these results were due to an additional program supporting pupils with motor deficits and a longer observation time of nine years. However, study length seems to play only a minor role as 12 weeks observation time was enough to report higher motor performance of girls in a school-based intervention [29].

It has to be questioned whether the baseline level of motor performance which was already higher in the OG could have influenced the potential for improvement. Prior studies revealing that a higher level of training status may be related with smaller improvements [30]. A previous review found out that children with a low baseline cardiorespiratory fitness benefited most of physical activity interventions [18]. Due to the overload principle it is somehow evident, that children with higher level of sports competence would need a certain greater increase of training intensity and frequency [31].

Three potential reasons could explain the higher baseline performance of the OG. Firstly, already two years before the study, pupils of OG were faced with five PE lessons weekly, while CG had only four. To our knowledge there are no dose response studies available which address whether already one additional PE lesson provided in a younger age group could explain this difference in motor performance. It is often argued that the age span between 6 and 12 years are critical for motor skill learning, however well-designed studies are scarce and more recent studies do not provide clear evidence that learning curves differ dramatically between age groups [32]. However, besides biological factors also socio-economic environment might have had different influences on groups as it has been shown that motor performance is influenced by home environment, nutritional supply and socio-economic factors [33]. To the best of our knowledge, we tried to avoid this possible bias, as schools of the CG were chosen by comparable surroundings to OGs schools such as infrastructure and neighbourhood of the school. Finally, adolescents with higher interest in sports and hence a higher fitness level may have been more attracted by the school trial which provided a higher amount of PE lessons. To minimize this bias, a school without an entry examination in sports and without a specific focus on sports in the content of its curriculum has been chosen for OG.

Furthermore, it is speculative that more motivated children not only chose a school with more PE lessons but also show a higher amount of physical activity in their leisure time. But there was no difference between OG and CG concerning the fulfilment of physical activity recommendations beside from movement time in PE lessons and sports clubs. Therefore, it can be suggested that movement behaviour in leisure time was comparable and did not influence motor performance differently in OG and CG. Long-term effects from earlier sports related experiences may have an effect as well. The findings of Lahti et al. [6] support this suggestion as a more physical active lifestyle has been found still four years after a school intervention with daily PE lessons.

### Gender aspects

Another important finding was that girls showed significant stronger improvement of motor performance than boys. This finding applies for CG as well as for OG, but at a closer look it is evident that CG girls had the highest elevation of motor performance, starting with the lowest and finishing with the highest scores compared to all the others. Our findings support the suggestion that girls enter the second open window for developing motor performance at a younger age, as their adolescence spurt starts in average two years earlier than in boys [34]. However, it is in contrast to several other studies reporting either no gender differences in improvement of motor performance or even better developments in boys [11, 35]. In a previous study motor performance of female adolescents correlated even negatively with their age and the authors explained it by decreasing time being physical active [36]. Anyway, as studies results are diverse a final conclusion cannot be drawn.

### Sports club membership

The current study found out that members of sports clubs outreached non-members in motor performance irrespective of the number of PE lessons provided in school, whereby number of sports club membership was similar between groups. It has been shown that sports club members are more physically active than non-members [37] potentially leading to higher motor performance. Furthermore, it can be suggested that more frequent PE lessons do not compete with sports club participation which is also supported by previous observations [5, 38], although controversial results exist [39]. Consequently, training in sports clubs is suggested to form an effective complement to PE lessons [2].

### Teaching content and quality

Beside the quantity of PE lessons, the selection of teaching content as well as quality of teaching can have a large influence on development of motor performance [3, 40]. In this study, teachers were asked to rank the motor ability they have been most focusing on during the whole observation period. In CG all the motor abilities, which teachers were mainly focusing on (strength, speed, and agility) notably increased. On the other hand, OG’s teachers planned to improve endurance, which is not reflected by the outcomes. These results provide further support for the suggestion that the focus of teaching content in combination with the selection of exercises plays an important role in the motor performance [29, 41]. Although endurance in OG was better at baseline, it decreased during the observation period. These findings go along with a five-month intervention study where aerobic capacity decreased in two intervention groups with different training programs and in CG. The authors of the previous study suggested that contents of the intervention programs and of PE lessons were not extensive enough to improve cardiorespiratory fitness [42]. Therefore, in the present study the content of the PE lessons in OG may not have been specific enough to enhance endurance, but also other motor abilities [43]. It can thus be suggested that a higher development of several motor abilities is less based on quantity, but more on quality of PE lessons (e.g. teaching methods, selection of exercises).

Further, the contents of the PE curriculum are of importance to discuss the present findings. The Austrian standard curriculum has the aim to develop condition and coordination abilities while promoting enjoyment in sports. This might explain why results of motor abilities differed between exercises. A more fitness-based PE curriculum might improve motor performance more likely and therefore, adaptations of the curriculum might be considered.

Pupils spend a lot of time sedentary in school and studying at home, therefore time for physical activity in school or sports clubs is limited and should be applied carefully. Enjoyable, effective and easy to practice training strategies like small-sided games are therefore preferred. Previous research [44] found out that young soccer players improved their technical ability and agility to a higher extend and with greater enjoyment when practicing small-sided games compared to a high-intensity training regime. On the other hand, the high-intensity training was more effective for speed-based conditioning. These findings show that PE content and method of training influence which abilities are supported most. Anyway, the importance of enjoyment of physical activity must not be neglected in time-efficient training contents of PE, even if this means that some abilities are less promoted than others [44]. The importance of adequate selection of exercises which are enjoyable and appropriate for age and abilities is recently confirmed by guidelines of the WHO [45]. Further work is required to establish the quality of PE lessons.

Finally, it must be mentioned that increasing physical active time is of importance not only for the improvement of motor performance but also for introducing a healthier lifestyle in youth. As school-based programs are unlikely to impact youth´s physical activity time without any support by their environment [38, 46], intersectional collaborations through policy, business, transport agencies and others are recommendable [3, 40].

### Limitations

Besides the observational character of the study which has its specific strength and weaknesses [47] some other issues should be mentioned here. One source of weakness in this study which could have affected the measurements of motor performance was the use of the GMT. The informative value of motor performance testing-tools is somehow limited as it may appear that participants are not able to transfer new motor abilities on the especially questioned exercise or cannot proof their abilities in competitive situations. Additionally, different sample sizes have been used when proofing the reference values of the GMT. In the value-tests for the exercises a different sample size has been used for each test, reaching form n = 150 to n = 12,000. Interestingly, when exercise-values were calculated with smaller sample sizes there is a higher improvement of motor performance in the present study compared to reference values. Anyway, this should not have influenced the comparison between OG and CG and hence this limitation seems not to be relevant for the interpretation of the primary research question of the current study. Motor performance has been assessed in the awareness of the difference between motor ability and motor performance. When observing an improvement on motor ability, adolescents are also asked to show if they are able to transfer it on the questioned exercise [48]. Due to the interaction between physical fitness and motor ability [49] the measurement of motor performance using the GMT might be influenced by the physical fitness of children. However, collecting data through the GMT, performed under similar conditions is widely spread across the scientific world to collect information on the improvement of motor performance [50, 51].

Further, the different baseline values in motor performance could potentially explain the results. Additionally, a two-way mixed ANOVA with baseline values used as covariates has been calculated. However, there was no different outcome compared to the presented calculations.

Measuring physical activity time self-reported by a questionnaire might be limited as participants sometimes tend to give socially desirable answers. Anyway, if participants may overestimate their physical activity time, this would apply for both groups. Consequently, comparability of OG and CG is ensured.

As summarized in a world-wide survey of school PE of the United Nations Educational, Scientific and Cultural Organization, PE curricular time allocation varies considerably even in Europe ranging from 30 to 290 min per week. Also, PE curricular themes (health-related fitness, motor ability, active lifestyle, personal and social development) are ranked differently. Therefore, data might be confined to countries/schools with a similar setting (described in the methods section) which makes generalisation to other settings difficult [52].

## Conclusions

Taken together these findings do not support the notion that a general increase of the amount of PE lessons would improve motor abilities. The results could be interpreted such that OG reached a potential “ceiling effect” (i.e., a limit in what could be achieved by training over time) at the beginning of the observation period, while CG required more time to reach the same level. Anyway, they give cause to move the debate forward to highlight the importance of the teaching content for specific target groups. It seems recommendable to focus on specific intervention programs with particular teaching contents to improve motor performance.

## Supporting information

S1 File Strobe-checklist. Checklist of items that should be included in reports of case-control studies.
(PDF)

S2 File Raw data. (XLSX)

S3 File Study protocol ethics committee—English. Application form for the assessment of a planned scientific study by the ethics committee of the University of Vienna.
(PDF)

S4 File Study protocol ethics committee—German. Application form for the assessment of a planned scientific study by the ethics committee of the University of Vienna.
(PDF)

S5 File Trend checklist. Reporting Checkliste for nonrandomized evaluations.
(PDF)
