# Supplementary material for: Influence of an increased number of physical education lessons on the motor performance of adolescents–A non-interventional cohort study
Source: PLoS One. 2021 Oct 14;16(10):e0258305. doi: 10.1371/journal.pone.0258305 (PMC8516264; doi:10.1371/journal.pone.0258305)
Supplement: S3 File — Application form for the assessment of a planned scientific study by the ethics committee of the University of Vienna. (PDF) [file pone.0258305.s003.pdf]

# Application form for the assessment of a planned scientific study by the ethics committee of the University of Vienna

## 1. Brief information on the planned study

### 1.1. Study title

Evaluation of an increased number of physical education lessons – an investigation of motor ability and self-esteem in Upper Austrian schools in the 7th and 8th grade

### 1.4. Brief description of the planned study (max. 500 words)

The present dissertation is a study that aims to observe the development of motor ability and self-esteem in children over a period of 1.5 years. The children are divided into two groups. Group A receives three lessons (50 minutes each) of physical education per week as intended in regular school curriculum. Physical education takes place twice a week because once a week two lessons are combined (100 minutes). This group (control group) consists of four classes (schools: [redacted]).
In addition, the development of motor ability and self-esteem in group B, which consists of three classes per week, is observed. These classes receive five lessons of 50 minutes each of physical education lessons per week included in their time table. These physical education classes take place four times a week, as two lessons are blocked one after the other.

Group A: 3 physical education lessons in school lessons (150 minutes).
Physical education lessons twice a week.
Group B: 5 physical education lessons in school lessons (250 minutes).
Physical education lessons four times a week.

No additional physical education lessons or planned changes in content are carried out as an intervention. The observation relates on the regular physical education lessons, which are differently organized in schools. We intend to analyze these circumstances of a different number of PE lessons more closely. The development of the motor ability will be observed under these different school timetables.
The motor ability is examined using the German motor ability test in the gym of the schools (see 1.7.). The self-esteem of the children is assessed with a widely used, standardized questionnaire (ALS - the list of statements on self-esteem for children and adolescents) in the pupils' classroom. One of the aims is to analyze whether there is a relation between high motor ability and high self-esteem in the children. Movement behavior in leisure time is also recorded to analyse its influence on motor ability. Since it is assumed that the children's movement behavior in leisure time varies depending on the season, the movement behavior is recorded using a questionnaire at each of the three test times.

The tests of motor ability and self-esteem are carried out three times: at the beginning of the summer semester 2017, at the beginning of the winter semester 2017/18 and in the summer semester 2018. A total of 150 test persons aged 12-14 years participates at the study.

### 1.5. Objectives of the study (questions, hypotheses, etc.)

The aim is to observe the development of motor ability and self-esteem in pupils with three or five physical education lessons per week over a period of three school semesters. Due to the increased activity time, a higher increase in motor ability is expected in children with five physical education lessons per week. As a result, it is examined whether an increase in motor ability is related to an increase on physical education lessons. It is unknown how the physical activity behavior of children outside of school is shaped as it may influence the development of motor ability and must therefore be recorded. It is possible that children with more lessons of physical education at school, do less physical activity in their free time and also have less club membership. This is recorded using a questionnaire. Furthermore, a possible change in self-esteem of the students is evaluated. The aim is

to determine whether an increase in motor ability is related to a change in self-esteem and whether students with good motor ability have a better self-esteem.

H1.1: In children which have an increased number of physical education lessons due to regular school curriculum, there is a higher development in motor ability (through an observation period of 1.5 years).

H1.2: There is a correlation between children's high self-esteem and children with good motor ability.

**1.6. Description of the study design (e.g. timepoint of survey, control groups, number of groups, sample size estimation, sampling, etc.)**

Samples were recruited by asking the school management of the respective schools. The approval of the Provincial School Board of Upper Austria contributed the acquisition of the selected school sample and gave recommendations to the selection for the recruiting.
Number of samples might be smaller due to missing agreed consent of parents or guardians, or due to injury or illness. Around 60 children in the test group and around 90 children in the control group will be recruited. The control group consists of children of two different schools, [redacted] and [redacted]. The experimental group from three classes of School [redacted].
The timepoints of testing are planned for the beginning of the 2017 summer semester, the 2017/18 winter semester and the 2018 summer semester. The children in the samples are in the 7th grade at the first and in the 8th grade at the second and third timepoint of testing.
The physical development of the students is a constant influencing factor and the pace of development might vary from student to student. To determine the biological age and thus to the physical development would mean confronting the children with a medical examination, which records, for example, the development of breast and pubic hair. These medical examinations cannot be carried out within the study as the children should not be exposed to such intimate examinations and the expenditure of time. The examination of the carpal bone by an X-ray is also considered a reliable way of determining the biological age and the level of physiological development. However, it is not reasonable for the test persons to expose themselves to radiation exposure for the desired data. The comparison of costs and benefits was reconsidered by the study management and found to be inadequate. It is also assumed that the number of samples drops sharply if children and legal guardians refuse the participation to the study to avoid this confrontation. Therefore, to avoid this expected fluctuation and still enable research in this important stage of development, it was decided to include the growth processes solely by recording body size.

**1.7. Description of the method of data collection, the sample (study participants), the study materials (e.g. instruments used) and the like.**

The data on motor ability are collected in the gym of the respective school, the self-esteem in a classroom and further evaluated using the SPSS statistical program.
The instruments used to assess self-esteem is the ALS test (the list of statements on self-esteem for children and adolescents). The German Motor ability Test will also be used; most of the materials required are available at the respective school: a long bench, a gym with floor markings for indoor volleyball, a gym mat and huts. A measuring tape, a light barrier and a balancing beam are owned by the test management.
More information about the method:
No additional exercise lessons or content-related interventions are carried out. The observation relates to the regular physical education lessons that are held differently in schools and is intended to analyze the resulting circumstances more closely. The development of motor ability of the different school conditions is analyzed.
The initial survey is used to analyze the start time of the observation period. The aim is not a similar level, but to analyze the change within the observation period of 1.5 years. Since children are exposed to different influences on physical activity behavior from early childhood (physical activity

on the way to school, content of physical education in elementary school, physical activity promotion in kindergarten, etc.) it is not possible to establish a clear, comparable initial situation.
The time of the start of observation was set so that the number of physical education lessons in school lessons is the largest difference in the lower grades between group A and B.
The measurement at the beginning of the 5th grade (where the increased number of physical education lessons in group A starts) was not selected because the number of physical education lessons in group B differs by only one unit at this point in time. The slight difference of 50 minutes in the 5th and 6th grades is therefore neglected. The observation time of the development of motor ability is put on the 7th and 8th grade. During these two school years, the number of physical education lessons differs by 100 minutes per week.
The starting level is analyzed at the beginning of the observation period. This is to prevent the competencies of the students from being compared, but rather their development in the observation period. It is not the "ability" but the change in performance that is analyzed.

**1.8. Planned start and expected total duration of the study**
March 2017; Total duration 36 months

**2. Study participants**
**2a Recruitment of participants and inclusion and exclusion criteria for participation in the study**

**2.1. Planned number of participants**
N = 150 participants who were tested at 3 timepoints

**2.2. Expected duration of study participation for the participants**
70 minutes per timepoint, total per person for all three timepoints 3.5 hours

**2.3. Characterization of the participants**
* Minimum age: 12 years Maximum age: 14 years
* Gender: male and female
* Are participants included which are not able to give consent personally? Yes
* Are the participants children, guardians or other vulnerable groups? Yes, children

**2.4. Describe the planned recruitment process (please enclose all materials intended for use, e.g. advertisements):**
The sample was recruited by asking respective school managements, as well as with the sports teachers of the school. The schools were selected based on the information of the state school board. (For more information on recruitment, see 1.6.) Both, legal guardians and students, can decide whether to participate to the study, reject or withdraw it in written and verbal form. It is not necessary for the class to take part in the test as a group, so each test person and his/her legal guardian can decide whether to participate or not.

**2.5. Briefly describe the selection of participants and the inclusion and exclusion criteria (if applicable: explicit justification for the inclusion of people from protected groups, e.g. minors, temporarily or permanently incapable of consent)**
The test subjects are minors at the age of 12-14 years. The inclusion criterion is attendance at one of the three selected schools (see 1.6.). Exclusion criteria are injuries and illnesses that do not allow physical exercise in the context of school sports lessons. It is not possible to take part in the surveys without the consent of the legal guardian. The signature of the participating students on the declaration of consent is also essential.
