# Supplementary material for: Influence of an increased number of physical education lessons on the motor performance of adolescents–A non-interventional cohort study
Source: PLoS One. 2021 Oct 14;16(10):e0258305. doi: 10.1371/journal.pone.0258305 (PMC8516264; doi:10.1371/journal.pone.0258305)
Supplement: S4 File — Application form for the assessment of a planned scientific study by the ethics committee of the University of Vienna. (PDF) [file pone.0258305.s004.pdf]

Antragsformular zur Beurteilung einer geplanten wissenschaftlichen Studie durch die Ethikkommission der Universität Wien

Version 13 (Oktober 2016)

Die beantragte Studie ist als Beilage anzuhängen. Es kann **zusätzlich** (!) zur kurzen Beantwortung der Fragen (max. 150 Wörter) auf den sich beziehenden Teil im Antragsformular verwiesen werden.

Nicht zutreffende Punkte (vor allem bei Untersuchungen an Tieren) bitte mit t.n.z. beantworten.

1. Allgemeines

1.1. Bei der vorgelegten Studie handelt es sich um:

☐ ein drittmittelfinanziertes Projekt

Fördergeber/Universitätsinterne Projektnummer/Kostenstelle (falls bereits vorhanden):

☐ ein universitätsinternes Projekt

☒ eine Dissertation (PhD-Arbeit)

Name des Betreuers/der Betreuerin:

XXXX

Die/der BetreuerIn wurde über die Einreichung informiert

☒ Ja ☐ Nein

☐ eine Masterarbeit/eine Diplomarbeit

Name der Betreuerin/des Betreuers:

Einreichung wurde beantragt von:

☐ Betreuerin/Betreuer ☐ Studienrechtliches Organ

1.2. Warum bzw. wofür wird ein Votum der Ethikkommission benötigt?

☐ Die Untersuchung könnte die physische oder psychische Integrität, das Recht auf Privatsphäre oder sonstige subjektive Rechte oder überwiegende Interessen von Versuchspersonen beeinträchtigen.

☒ Das Votum wird von einem Publikationsorgan verlangt.

☐ Das Votum wird von einem Fördergeber verlangt.

☐ Sonstige Gründe, nämlich:

1.3. Wurde für die Studie bereits ein Begutachtungsverfahren durch eine Ethikkommission durchgeführt?

☐ Ja ☒ Nein Wenn Ja, Gutachten beilegen!

2. Kurzinformationen zur geplanten Studie

2.1. Titel der Studie

Evaluation einer erhöhten Anzahl an Sport- und Bewegungseinheiten – Untersuchung der sportmotorischen Fähigkeiten und des Selbstwertgefühls an Oberösterreichischen Schulen in der 7. und 8. Schulstufe

2.2. Handelt es sich um eine Teilstudie innerhalb eines größeren Forschungsvorhabens? Wenn ja: bitte den Titel dieses größeren Projekts angeben!

Nein

2.3. Fachdisziplin

Sportsoziologie

2.4. Kurzbeschreibung der geplanten Studie (max. 500 Wörter)

Bei der vorliegenden Dissertation handelt es sich um eine Studie, welche die Entwicklung der sportmotorischen Fähigkeiten und des Selbstwertgefühls von Kindern in einem Zeitraum von 1,5 Jahren beobachten soll. Die Kinder sind in zwei Gruppen geteilt. Gruppe A erhält drei Einheiten (je 50 Minuten) Sport- und Bewegungsunterricht wöchentlich im Rahmen des Regelschulunterrichts. Der Sportunterricht findet zwei Mal wöchentlich statt, da an einem Wochentag zwei Einheiten zusammengelegt sind (Doppelstunde 100 Minuten). Diese Gruppe setzt sich aus vier Klassen zusammen (Schulen: XXX).
Des weiteren wird die Entwicklung der sportmotorischen Fähigkeiten und des Selbstwertgefühls von Gruppe B beobachtet, welche aus drei Klassen besteht. Diese Klassen erhalten wöchentlich fünf Einheiten zu je 50 Minuten Sport- und Bewegungsunterricht im Rahmen des Schulunterrichts. Wöchentlich findet dieser Sportunterricht viermal statt, da je einmal zwei Einheiten hintereinander geblockt sind.

Gruppe A: 3 Sport- und Bewegungseinheiten im Schulunterricht (150 Minuten).
Es findet zwei Mal wöchentlich Sport- und Bewegungsunterricht statt.
Gruppe B: 5 Sport- und Bewegungseinheiten im Schulunterricht (250 Minuten).
Es findet vier Mal wöchentlich Sport- und Bewegungsunterricht statt.

Es werden keine zusätzlichen Bewegungseinheiten oder gezielte inhaltliche Intervention durchgeführt. Die Beobachtung bezieht sich auf den an den Schulen unterschiedlich gestalteten regulären Sportunterricht und soll die dadurch enstehenden Umstände genauer analysieren. Es soll die Entwicklung der sportmotorischen Fähigkeiten unter diesen unterschiedlichen, schulischen Umständen beobachtet werden.

Die sportmotorische Entwicklung wird anhand des Deutschen Motorik-Tests im Turnsaal der jeweiligen Schulen untersucht (siehe 2.8.). Das Selbstwertgefühl der Kinder wird anhand eines vielverwendeten, standardisierten Fragebogen im Klassenzimmer der Schüler/innen erhoben (ALS- Die Aussagenliste zum Selbstwertgefühl für Kinder und Jugendliche). Im weiteren vorgehen soll analysiert werden, ob ein Zusammenhang zwischen hoher sportmotorischer Fähigkeit und einem hohen Selbstwertgefühl der Kinder besteht. Auch das Bewegungsverhalten in der Freizeit wird aufgenommen um diesen Einflussfaktor auf die Sportmotorik zu ergänzen. Da vermutet wird, dass je nach Jahreszeit eine unterschiedliche Gestaltung des Freitzeitverhaltens der Kinder besteht, wird das Bewegungsverhalten per Fragebogen zu jedem der drei Testungszeitpunkten aufgenommen.

Die Erhebung der sportmotorischen Fähigkeit und auch des Selbstwertgefühls wird drei Mal durchgeführt, zu Beginn des Sommersemesters 2017, zu Beginn des Wintersemesters 2017/18 und im Sommersemester 2018. Für die Studie werden gesamt 150 Proband/inn/en im Alter von 12-14 Jahren getestet.

2.5. Zielsetzung der Studie (Fragestellungen, Hypothesen etc.)

Es soll eine Beobachtung zur Entwicklung der sportmotorischen Fähigkeiten und des Selbstwertgefühls von Schüler/innen mit wöchentlich drei bzw. fünf Sport- und Bewegungseinheiten im regel Schulunterricht in einem Zeitraum von drei Schulsemestern erfasst werden. Aufgrund der vermehrten sportlichen Aktivität wird eine höhere Steigerung der sportmotorischen Fähigkeiten bei Kindern mit fünf Einheiten Sport- und Bewegungsunterricht wöchentlich erwartet. Infolge dessen wird untersucht, ob eine Steigerung der sportmotorischen Fähikgkeiten in Zusammenhang mit einer Erhöhung der sportmotorischen Fähigkeiten steht. Dabei ergibt sich die Fragestellung, wie sich das Bewegungsverhalten der Kinder außerhalb der Schule gestaltet. Es ist ebenfalls ein Einflussfsktor auf die Entwicklung der sportmotorischen Fähigkeiten und muss deshalb beachtet und erfasst werden. Es besteht die Möglichkeit, dass Kinder mit erhöhter Bewegungszeit in der Schule, weniger Sport in der Freizeit ausüben und auch weniger Vereinszugehörigkeit aufweisen. Dies wird anhand eines Fragebogens überprüft. Weiters wird auf eine Veränderung des Selbstwertgefühls der Schüler/innen geachtet. Es soll erhoben werden, ob eine Steigerung der sportmotorischen Fähigkeiten in Zusammenhang mit einer Veränderung des Selbstwertgefühls steht und ob Schüler/innen mit guten sportmotorischen Fähigkeiten ein besseres Selbstwertgefühl aufweisen.

H1: Bei Kindern mit einer erhöhten Anzahl an Sport- und Bewegungseinheiten im regulären Schulunterricht besteht eine höhere Steigerung der sportmotorischen Fähigkeiten (im Laufe der Beobachtungszeit von 1,5 Jahren).

H1: Es besteht ein Zusammenhang zwischen Kindern mit einem hohen generalisierten Selbstwertgefühl und Kindern mit guten sportmotorischen Fähigkeiten.

2.6. Wissenschaftliche und gesellschaftliche Relevanz der Studie

Die Studie soll zeigen, ob die Einführung einer erhöhten Anzahl an Sport- und Bewegungseinheiten einen Einfluss auf die sportmotorischen Fähigkeiten von Kindern hat. Es wird angestrebt in Erfahrung zu bringen, ob das Selbstwertgefühl der Kinder durch höhere sportmotorische Fähigkeiten beeinflusst wird, was für die einzelnen Kinder eine Auswirkung auf ein zufriedeneres Zusammenleben in der Gesellschaft bringen kann. So könnte infolge des Studienergebnisses eine Empfehlung zu einer Erhöhung der Anzahl an Sport- und Bewegungseinheiten in der Schule für eine Verbesserung der sportmotorischen Fähigkeiten abgegeben werden.

2.7. Beschreibung des Untersuchungsdesigns (z. B. Erhebungszeitpunkte, Kontrollgruppen, Anzahl der Gruppen, Fallzahlschätzung, Stichprobengewinnung u. dgl.)

Die Stichprobengewinnung erfolgte im Vorfeld durch die Anfrage bei der Schulleitung der jeweiligen Schulen. Auch die Genehmigung des Landesschulrats Oberösterreich erbrachte einen wesentlichen Anteil an der Gewinnung der ausgewählten Stichprobe. Durch dessen Empfehlung wurde diese Auswahl an Schulen getroffen.

Bei nicht Einstimmung der Erziehungsberechtigten sowie durch Verletzungen oder Krankheit kann die Anzahl der Stichprobe kleiner ausfallen. Es sollen ca. 60 Kinder der Versuchsgruppe und ca. 90 Kinder der

Kontrollgruppe getestet werden. Die Kontrollgruppe besteht aus den Kindern zweier verschiedener Schulen, XXX und XXX. Die Versuchsgruppe wird aus den Klassen der Schule XXX geformt.
Die Erhebungszeitpunkte sind jeweils zu Semesterbeginn des Sommersemesters 2017, Wintersemester 2017/18 und des Sommersemester 2018 geplant. Die Kinder der Stichproben befinden sich zum Zeitpunkt der ersten Testung in der 7. Schulstufe und zu den beiden zweiten Testzeitpunkten in der 8. Schulstufe.

Die körperliche Entwicklung der Schüler/innen ist ein ständig begleitender Einflussfaktor und verläuft bei den Schüler/inne/n unterschiedlich schnell. Das biologische Alter zu bestimmen und somit ihre körperliche Entwicklung festzustellen würde bedeuten die Kinder einer medizinischen Untersuchung zu unterziehen, welche beispielsweise Brustansatz und Schambehaarung aufnimmt. Im Rahmen der Studie besteht keine Möglichkeit diese medizinischen Komponenten zu Untersuchung. Die Kinder sollen dem Zeitaufwand und dem Eingriff in solch intime Untersuchungen nicht ausgesetzt werden. Auch die Untersuchung des Handwurzelknochens durch ein Röntgenbild wird als verlässliche Form gewertet um das biologische Alter und somit den Entwicklungsstand der Kinder zu bestimmen. Es ist allerdings den Proband/inn/en nicht zumutbar sich einer Strahlenbelastung auszusetzen um die gewünschten Daten zu erhalten. Die Gegenüberstellung von Kosten und Nutzen wurde von der Studienleitung überdacht und als nicht adäquat empfunden. Weiters wird vermutet, dass die Stichprobenanzahl stark sinkt, wenn Kinder und Erziehungsberechtigte dieser Konfrontation aus dem Weg gehen und die Teilnahme an der Studie verweigern. Um dies zu verhindern und trotzdem eine Forschung in dieser für die sportmotorischen Fähigkeiten wichtigen Entwicklungsstufe zu ermöglichen, wurde entschieden, die Wachstumsprozesse allein anhand der Körpergröße einzubeziehen.

2.8. Beschreibung der Methode der Datenerhebung, der Stichprobe (StudienteilnehmerInnen), der Studienmaterialien (z. B. verwendete Instrumente) u. dgl.

Die Daten der sportmotorischen Fähigkeiten werden im jeweiligen Turnsaal der Schule und das Selbstwertgefühl in einer Schulklasse erhoben und weiters anhand des Statistikprogramms SPSS ausgewertet.
Unter den verwendeten Instrumenten befindet sich der ALS-Test (Die Aussagen-Liste zum Selbstwertgefühl für Kinder und Jugendliche). Weiters wird der Deutsche Motorik-Test angewandt, die benötigten Materialien sind zum Großteil an der jeweiligen Schule vorhanden. Benötigt wird eine Langbank, ein Turnsaal mit Bodenmarkierungen des Hallenvolleyballfeldes, eine Turnmatte und Hüttchen zur Bodenmarkierung. Ein Maßband, ein Lichtschranken und ein Balancierbalken werden von der Testleitung mitgebracht.

Weitere Informationen zur Methode:
Es werden keine zusätzlichen Bewegungseinheiten oder gezielte inhaltliche Intervention durchgeführt. Die Beobachtung bezieht sich auf den an den Schulen unterschiedlich oft gehaltenen regulären Sportunterricht und soll die dadurch entstehenden Umstände genauer analysieren. Die Entwicklung der sportmotorischen Fähigkeiten unter diesen unterschiedlichen, schulischen Umständen wird dabei analysiert.
Die Eingangserhebung dient um den Startzeitpunkt des Beobachtungszeitraumes zu analysieren. Nicht ein gleiches Ausgangsniveau ist anzustreben, sondern die Veränderung innerhalb des Beobachtungszeitraumes von 1,5 Jahren zu analysieren. Da die Kinder schon von Kleinkindalter an unterschiedlichen Einflüssen auf das Bewegungsverhalten ausgesetzt sind, (aktive Gestaltung des

Schulweges, Inhalt des Sportunterrichts in der Volksschule, Bewegungsförderung im Kindergarten etc.) ist es nicht möglich eine eindeutige, sich ähnelnde Ausgangssituation festzusetzen.
Der Zeitpunkt des Beobachtungsbeginns wurde so gesetzt, dass die Anzahl an Sport- und Bewegungseinheiten im Schulunterricht den größten Unterschied in der Unterstufenzeit zwischen Gruppe A und B beträgt.
Die Messung zu Beginn der 1. Klasse AHS (wo die erhöhte Anzahl an Sport- und Bewegungseinheiten der Gruppe A startet) wurde nicht gewählt, da sich die Anzahl an Sport- und Bewegungseinheiten zu Gruppe B zu diesem Zeitpunkt um nur eine Einheit unterscheidet. Der geringe Unterschied von 50 Minuten in der 1. und 2. Klasse Unterstufe (5. u 6. Schulstufe) wird deshalb vernachlässigt. Die Beobachtungszeit der Entwicklung der Sportmotorik konzentriert sich auf die 3. und 4. Klasse (7. u 8. Schulstufe). In diesen beiden Schuljahren unterscheidet sich die Anzahl an Sport- und Bewegungseinheiten um 100 Minuten wöchentlich.
Zu Beginn des Beobachtungszeitraumes wird das Ausgangsniveau analysiert. Dies soll verhindern, dass nicht die Kompetenzen der Schüler/innen verglichen werden, sondern ihre Entwicklung im Beobachtungszeitraum. Nicht ihr „Können“ sondern ihre Leistungsveränderung wird dabei analysiert.

2.9. Geplanter Beginn und voraussichtliche Gesamtdauer der Studie

März 2017; Gesamtdauer 36 Monate

3. StudienteilnehmerInnen

3a Rekrutierung der Teilnehmenden sowie Ein- und Ausschlusskriterien für die Studienteilnahme

3.1. Geplante Anzahl der Teilnehmenden

N=150 Personen die an 3 Erhebungszeitpunkten getestet werden

3.2. Voraussichtliche Zeitdauer der Studienteilnahme für die Teilnehmenden

70 min pro Erhebungszeitpunkt, gesamt pro Person für alle drei Erhebungszeitpunkte 3,5h

3.3. Charakterisierung der Teilnehmenden

- Mindestalter: 12 Jahre Höchstalter: 14 Jahre
- Geschlecht: ☒ männlich ☒ weiblich
- Sind nicht persönlich Einwilligungsfähige einschließbar? ☒ Ja ☐ Nein
- Handelt es sich bei den Teilnehmenden um Kinder, Besachwaltete oder andere vulnerable Gruppen?

Ja, es handelt sich um Kinder.

3.4. Beschreiben Sie das geplante Rekrutierungsverfahren (bitte alle zur Verwendung bestimmten Materialien, z. B. Inserate, beilegen):

Die Rekrutierung erfolgte anhand Telefonaten mit der jeweiligen Schulleitung, sowie mit den Sportlehrer/innen der Schule. Die Schulen wurden anhand der Empfehlung des Landesschulrats ausgewählt. (weiteres zur Rekrutierung siehe 2.7.) Sowohl Erziehungsberechtigte als auch die Schüler/innen können frei über die Teilnahme an der Studie entscheiden und diese in schriftlicher und mündlicher Form ablehnen bzw. zurückziehen. Es ist nicht erforderlich, dass die Klasse geschlossen an der

Testung teilnimmt, somit kann jede/r Proband/in und dessen Erziehungsberechtigte frei über die Teilnahme entscheiden.

3.5. Legen Sie kurz die Auswahl der Teilnehmenden sowie die Ein- und Ausschlusskriterien dar (wenn zutreffend: explizite Begründung für den Einschluss von Personen aus geschützten Gruppen, z. B. Minderjährigen, temporär oder permanent nicht einwilligungsfähigen Personen)

Die Proband/innen sind minderjährige Personen, welche sich im Alter von 12-14 Jahren befinden. Einschlusskriterium ist der Besuch einer der drei ausgewählten Schulen (siehe 2.7.). Ausschlusskriterien sind Verletzungen und Krankheiten die keine körperliche Ertüchtigung im Rahmen des Schulsportunterrichts erlauben. Die Teilnahme an den Erhebungen ohne ausdrückliche Einverständniserklärung der Erziehungsberechtigten ist nicht möglich. Auch die Unterschrift der teilnehmenden Schüler/innen in Form der Einverständniserklärung ist unumgänglich.

3.6. Wird die Zustimmung der Teilnehmenden oder deren gesetzlicher Vertretung eingeholt?

☒ Ja (Informationsblätter und Einwilligungserklärung beilegen)

☐ Nein Wenn nein, warum nicht:

3.7. Welche (persönlichen, sozialen, institutionellen) Beziehungen bestehen zwischen den Teilnehmenden und den Studiendurchführenden (z. B. Studierende-Lehrkraft, Dienstnehmer/in-Dienstgeber/in etc.)? Ist die Freiwilligkeit der Studienteilnahme gewährleistet?

Als Versuchsleiterin und Studienleiterin wird die Antragstellerin und als Studiendurchführende werden Studierende und Studienassistent/innen des Instituts für Sportwissenschaft der Universität Wien fungieren. Die Termine der Durchführung werden nach Vorlagen des Belegungsplans des Turnsaals der jeweiligen Schule zugeteilt und in Absprache mit den Sportlehrer/inne/n der Kinder eingeteilt. Es wird darauf geachtet, dass der Schulbetrieb von den Testungen nicht beeinflusst oder gestört wird. Die Freiwilligkeit der Teilnahme wird in allen Fällen betont und ausdrücklich im Rahmen der Einverständniserklärung hervorgehoben und erläutert. Die Teilnahme/Nicht Teilnahme oder der Abbruch der Erhebung auf Wunsch des/der Proband/en/in oder des/der Erziehungsberechtigten ist an keinerlei Konsequenzen vonseiten der Versuchsleitung bzw. der Studienleitung gebunden.

3b Datenschutz

3.8. Welche personenbezogenen Daten werden erhoben?

Name, Geschlecht, Geburtsdatum, Gewicht, Größe

3.9. Wie wird die Anonymität der Teilnehmenden gewährleistet?

Es werden keine Namen für die Auswertung verwendet, sondern Kennziffern. Die Zuordnung der Namen zu den dreistelligen Codes, mit denen alle weiteren Analyseschritte durchgeführt werden ist nur mittels der unterzeichneten Einverständniserklärung möglich. Die Einverständniserklärungen werden von Astrid Reif (Antragstellerin und Versuchleiterin) sicher verwahrt (versperrbarer Schrank), sind demnach nicht offen zugänglich und werden nicht digitalisiert oder übertragen. Die protokollierten Testdaten (Leistungsdaten & subjektive Bewertungsdaten) werden in das statistische Auswertungsprogramm SPSS/Statistica übertragen und am Institut für Sportwissenschaften durch Univ.-Ass. Mag. Astrid Reif

verwahrt. Der SPSS Datensatz wird auf einer Passwort-geschützten externen Festplatte gespeichert und nur für die Berrechnungen auf einem internetfähigen PC benutzt bzw. zwischengespeichert. Nach Abschluss der Berechnungen wird der Datensatz vom PC gelöscht und befindet sich wieder ausschließlich auf der Passwort-geschützten Festplatte. Im Rahmen der Auswertungen werden ausschließlich gruppenanalytische Fragestellungen berechnet und ggf. publizert. Einzelne Fallanalysen werden nicht publiziert. Ein Rückschluss auf einzelne Personen ist somit nicht möglich.

3.10. Wenn eine vollständige Anonymisierung personenbezogener Daten nicht möglich ist: Wie wird die Privatsphäre der Teilnehmenden geschützt?

Siehe 3.9.

3.11. Werden Stimmen, Bilder oder Videos aufgenommen?

☐ Ja ☒ Nein

Wenn Ja: Wird die Einwilligung der Teilnehmenden zur Aufnahme eingeholt?

3.12. Wie wird gewährleistet, dass die Teilnehmenden jederzeit die laufende Mitwirkung an der Studie abbrechen können?

Offenlegung der Möglichkeit zum Abbruch wird zu Beginn der Datenerhebung und anhand der Teilnehmer/inneninformation betont; Auf Wunsch der Proband/innen kann jederzeit abgebrochen werden.

3.13. Wie erfolgt die Verarbeitung und Auswertung der Studiendaten?

☐ personenbezogen, Begründung:

☒ indirekt personenbezogen Wie erfolgt die Anonymisierung?

Vercodierter Datensatz - Sicherung Datensatz auf passwortgeschützter Festplatte. In Datensatz erfolgt nur die Eintragung des Codes.

3.14. Wie wird mit den Studiendaten nach Abschluss der Studie umgegangen? (Aufbewahrung: wie, wo, für wen zugänglich, wie lange? Wenn Löschung: wann?).

Sicherung Datensatz auf passwortgeschützter Festplatte; Löschung nach 3 Jahren durch Univ.-Ass. Mag. Astrid Reif oder vorher auf Wunsch der Studienteilnehmer/innen.

3.15. Wie wird den Teilnehmenden die Einsicht in ihre persönlichen Studiendaten ermöglicht?

Inidividueller Termin wird via Email koordiniert und in persönlicher Besprechung mit Astrid Reif wird Einsicht in Datensatz geboten.

3.16. Können sich Teilnehmende über die Forschungsergebnisse informieren?

☒ Ja ☐ Nein Wenn nein, Begründung:

3.17. Auf welche Weise und wie lange können die Teilnehmenden die Löschung ihrer Studiendaten verlangen?

Via Email oder Telefonat - an Astrid Reif; 3 Jahre

3c Folgen der Studienteilnahme für die Teilnehmenden

3.18. Ist die Teilnahme an der Studie für die Teilnehmenden mit absehbaren Risiken oder anderen anzunehmenden problematischen Begleiterscheinungen verbunden (z.B.: Schmerzen, Unannehmlichkeiten oder Verletzungen der persönlichen Integrität)? Welche Maßnahmen werden zur Vermeidung und/oder Versorgung von unvorhergesehenen/unerwünschten Ereignissen getroffen?

Keine Risiken. Bestreben eines standardisierten Untersuchungsablaufes. Die gesetzten Belastungen sind für die Kinder nicht schädlich und überschreiten nicht die Anforderungen des Unterrichtes oder des freien Spielens. Somit ist die Testung für die Sicherheit der Kinder unbedenklich und es sind keine absehbaren Risiken mit dieser Testung verbunden.

3.19. Welche Verfahren werden eingesetzt, um unerwünschte Effekte einer Studienteilnahme zu identifizieren, diese zu dokumentieren und zu berichten? Beschreiben Sie, wann, durch wen und wie dies erfolgt, z. B. freies Befragen und/oder an Hand von Fragelisten.

Die Proband/inn/en können zu jederzeit bei der Erhebung der Daten von den Tester/inne/n und dem/der Sportlehrer/in kontaktiert werden und bei unerwünschten Effekten die Studie abbrechen. Eine Kontaktaufnahme zwischen der Projektleitung und den Schüler/inne/n bzw. Erziehungsberechtigten ist zu jeder Zeit über die am Teilnahmeinformationsblatt bekannt gegebenen Kontaktdaten oder über die/den jeweilige/n Klassenlehrer/in möglich.

3.20. Wird eine Teilnahmevergütung bzw. eine Aufwandsentschädigung (Ausgleich von Fahrtspesen und Einkommensentgang) an die Teilnehmenden bezahlt?

Nein

3.21. Welche voraussichtlichen Vorteile bzw. welcher mögliche Nutzen sind für die Teilnehmenden mit der Studie verbunden?

Keine

3d Weitere ethische Aspekte

3.22. Werden die Teilnehmenden in vollem Umfang über Art, Ziel und Inhalt der Studie informiert?

☒ Ja ☐ Nein, Begründung:

Werden die Teilnehmenden getäuscht?

☐ Ja ☒ Nein

Wenn ja: Beschreibung und Begründung der Notwendigkeit der Täuschung:

3.23. Welche weiteren möglicherweise auftretenden Probleme bzgl. Studienteilnahme und Studiendurchführung gibt es aus Ihrer Sicht?

keine

3.24. In welchem Verhältnis stehen potenzielle Risiken der Studie zum erwarteten wissenschaftlichen und gesellschaftlichen Nutzen?

keine gesundheitlichen Risiken

3.25. Unter welchen Bedingungen ist eine Unterbrechung der Studie vorgesehen? Unter welchen Umständen wird die Studie gänzlich abgebrochen?

Jede/r Teilnehmer/in kann jederzeit, aus welchen Gründen auch immer, die Teilnahme abbrechen. Die Studie wird abgebrochen, wenn sich alle Teilnehmer/innen dafür entscheiden nicht mehr an der Studie teilzunehmen.

4. Sonstige Anmerkungen

5. Studiendurchführende

Geben Sie alle an der Studie Mitarbeitenden an:

Bitte berücksichtigen Sie dabei ggf. die Abgrenzung der Funktionen Projektleitung (Leitung des Gesamtprojekts), Studienleitung (Leitung einer Teilstudie) und Versuchsleitung (Durchführung einer Studie). Diese unterschiedlichen Funktionen bzw. Zuständigkeiten sollen auch in der TeilnehmerInneninformation und Einverständniserklärung entsprechend abgebildet werden.

Name	Institution	Funktion*	Qualifikation**
Astrid Reif	Universität Wien, Institut für Sportwissenschaft	Versuchs- und Studienleitung	prae doc
XXXX	Universität Wien, Institut für Sportwissenschaft	Datenerhebung und Auswertung	Studienassistent
XXXX	Universität Wien, Institut für Sportwissenschaft	Datenerhebung und Auswertung	Studienassistentin
XXXX	Universität Wien, Institut für Sportwissenschaft	Datenerhebung und Auswertung	Studienassistent
XXXX	Universität Wien, Institut für Sportwissenschaft	Datenerhebung und Auswertung	Studienassistentin

* z. B. Studienleitung, Projektleitung, Versuchsleitung, Planung, Auswertung, Datenerhebung

** z. B. Senior Researcher, Dissertant/in, Postdoc usw.

Gibt es Interessenskonflikte zwischen den beteiligten Forscher(inne)n?

☐ Ja ☒ Nein

Wenn Ja: Beiblatt Interessenskonflikt ausfüllen und beilegen.

6. Name und Unterschrift der antragstellenden Person

Name: Astrid Reif
Institution/Firma: Universität Wien, Institut für Sportwissenschaft
Position: prae doc

Unterschrift der antragstellenden Person: Hiermit bestätige ich, dass die in diesem Antrag gemachten Angaben zur geplanten Studie korrekt sind und die Studie gemäß dieser Angaben und in Übereinstimmung mit den Prinzipien guten wissenschaftlichen Arbeitens durchgeführt werden wird.

Unterschrift der Antragstellerin/des Antragstellers, Datum

6.1. Zustellungsbevollmächtigte/r falls nicht antragstellende Person

